# Invasive Type e *Haemophilus influenzae* Disease in Italy

**DOI:** 10.3201/eid0902.020142

**Published:** 2003-02

**Authors:** Marina Cerquetti, Marta Luisa Ciofi degli Atti, Rita Cardines, Stefania Salmaso, Giovanna Renna, Paola Mastrantonio, the Hi Study Group

**Affiliations:** * Istituto Superiore di Sanità, Rome, Italy

**Keywords:** *Haemophilus influenzae*, invasive disease, adult, dispatch

## Abstract

We describe the first reported cases of invasive type e *Haemophilus influenzae* disease in Italy. All five cases occurred in adults. The isolates were susceptible to ampicillin and eight other antimicrobial agents. Molecular analysis showed two distinct type e strains circulating in Italy, both containing a single copy of the capsulation locus.

In Italy, the vaccine against *Haemophilus influenzae* type b (Hib) was licensed in 1995, but vaccination is voluntary. Vaccination coverage by 24 months of age was estimated in 1998 at 19.8% for the 1996 birth cohort (*1*). Coverage increased in 2000 to 53.1% for the 1998 birth cohort (*2*). Since 1994, surveillance of *H. influenzae* meningitis has been conducted within the National Surveillance of Bacterial Meningitis (*3*). A laboratory-based active surveillance of invasive *H. influenzae* disease was implemented in a sample of Italian regions in 1997 (*4*). In 1997–1998, the incidence of invasive Hib disease estimated by this system was lower than that reported in northern and central Europe and in the United States before mass vaccination was introduced, yet was comparable with the incidence reported in other Mediterranean countries with similar vaccination coverage (*4*).

In 1998–2001, laboratory-based active surveillance was conducted in seven Italian regions, including a population of approximately 24 million persons (33% of the Italian population). Participating regions were located throughout the country and included rural, urban, and large metropolitan areas. Active *H. influenzae* case finding was conducted by contacting, monthly, microbiologists from the regional laboratories of hospitals with infectious disease or pediatric wards. A patient with invasive disease was defined as a patient with a compatible illness, accompanied by isolation of *H. influenzae* from a normally sterile site or detection of Hib antigen in cerebrospinal fluid. Hospital microbiologists were asked to send *H. influenzae* isolates to the national reference laboratory at Istituto Superiore di Sanità, where all strains were assayed by polymerase chain reaction (PCR) capsular genotyping. Serotyping by slide agglutination was performed at the regional level, when possible, and at the national reference laboratory.

From 1998 to 2001, a total of 219 cases of invasive *H. influenzae* disease were reported; 165 were diagnosed by isolation of *H. influenzae* from a normally sterile site, and 54 were diagnosed by detection of Hib antigen in cerebrospinal fluid. Of the 165 isolates, 97 (58.8%) were sent to the national reference laboratory; the percentage of isolates sent to the reference laboratory remained relatively stable over the years (from 62.3% in 1998 to 58.3% in 2001). Analysis of incidence data by serotype showed that the annual number of cases of invasive Hib disease decreased from 69 in 1998 to 17 in 2001, while the number of nontypable *H. influenzae* remained constant (mean 8 cases/year; range 7–11) (Table 1). No cases attributable to capsulated *H. influenzae* other than type b were detected in years 1998–1999; in the next 2 years, a total of seven cases were traced; five were due to type e strain.

**Table 1:** Incidence per 100,000 persons and cases of invasive *Haemophilus influenzae* disease, by serotype, Italy, 1998–2001

	Incidence per 100,000 (no. of cases)			
*H. influenzae*	1998	1999	2000	2001
All capsular types plus nontypable^a^	0.35 (84)	0.26 (64)	0.16 (40)	0.13 (31)
Type b	0.28 (69)	0.18 (44)	0.09 (21)	0.07 (17)
Nontypable	0.05 (11)	0.03 (7)	0.03 (7)	0.03 (7)
Capsulated other than b	0 (0)	0 (0)	0.02 (4)	0.01 (3)

^a^Includes isolates that were not serotyped.

We describe these five cases of invasive disease caused by *H. influenzae* type e (Hie). Genetic relationship among the five Hie isolates was assessed by pulsed-field gel electrophoresis (PFGE). Susceptibility to nine antimicrobial agents, including ampicillin, was also determined. Because amplification of capsulation (*cap*) locus is assumed to contribute to strain virulence, the copy number of *cap* e locus in each isolate was also identified.

## The Study

Five Hie isolates were detected through surveillance from January 2000 to December 2001. Characteristics of patients were obtained by reviewing clinical records (Table 2). Briefly, two cases, both with meningitis, occurred in young adults, who recovered*.* The remaining three cases, two with bacteremic pneumonia and one with septicemia, were in elderly patients, who died. All but one (no. 2) of the patients were from neighboring regions in northeastern Italy. However, the towns they lived in were quite distant, and the patients were admitted to different hospitals in different periods. Serotyping of the isolates by slide agglutination was performed by using polyvalent and monovalent antisera to capsular serotypes a through f (Difco Laboratories, Detroit, MI). Capsular genotype was identified by PCR (*5*). Briefly, in a first round of PCR, primers to the *omp*P2 gene were used to confirm the *H. influenzae* species, while primers directed to the *bex* region confirmed capsulation. A second round of PCR with primers directed to the *cap* e–specific region (*6*) generated an expected product of 1,350 bp. Further characterization of the isolates was performed by phenotypic and genotypic methods. Biotypes were assigned by determining indole production, urease level, and ornithine decarboxylase activities. MICs of ampicillin were determined by E-test (AB Biodisk, Solna, Sweden). Hib strain ATCC 10211 was used as control. Susceptibilities to trimethoprim/sulfamethoxazole, ceftazidime, chloramphenicol, azithromycin, aztreonam, ciprofloxacin, imipenem, and tetracycline were tested by disk diffusion assay. Both the E-test and the disk diffusion assay were performed by using *Haemophilus* test medium, as recommended by the National Committee for Clinical Laboratory Standards (NCCLS) (*7*). Interpretative breakpoints and zone diameters were also based on NCCLS criteria. Production of β-lactamase was detected by the cefinase disk test (BBL, Becton-Dickinson, Sparks, MD). Restriction fragment length polymorphism analysis by PFGE was conducted as described (*5*), except for digestion of genomic DNA, performed by using *Sma*I (20 U) or *Apa*I (20 U) restriction enzymes (New England BioLabs, Wilbury Way Hitchin, U.K.). The copy number of *cap* e locus was determined by Southern blot analysis, using as probe the 1,350-bp amplicon obtained by PCR of a prototype type e strain. Since the *Kpn*I and *Sma*I sites flank the *cap* locus of encapsulated *H. influenzae* strains, the copy number of the locus can be estimated by the size of the restriction fragment obtained after digestion of the chromosome with these enzymes (*8*). Restriction fragments were separated by PFGE as described (*5*), transferred to nylon membranes, and hybridized with the probe. Labeling of the probe and hybridization reactions were obtained by using the ECL kit (Amersham Pharmacia Biotech, Little Chalfont, U.K.). Hybridizing bands were visualized by autoradiographs. Although no data are available on the size of *cap* e locus, on the basis of results obtained on Hib strains, the DNA fragment for a single-copy strain was expected to be approximately 27–28 kb. In fact, the *Kpn*I/S*ma*I fragment includes the *cap* locus, whose size is 18 kb in Hib strains, plus additional segments (about 10 kb) upstream and downstream of the *cap* region (*8*). Strains with two or more copies of the *cap* b locus featured fragments of increased size (45 kb, 63 kb, 81 kb, 99 kb) (*8*).

**Table 2:** Clinical data of patients with Hie invasive disease and characterization of isolates^a^

Patient no.	Mo/yr of onset	Age	Gender	Signs and symptoms	Underlying condition	Outcome	Site of isolation	Biotype	PFGE pattern	Copy no. of *cap* e locus
1	01/2000	75	M	Bacteremic pneumonia	Chronic lymphocytic leukemia	Died	Blood	IV	1a	1
2	05/2000	35	M	Meningitis	Head trauma from car accident	Survived	CSF	IV	2	1
3	10/2000	65	M	Septicemia	Retro-peritoneal sarcoma	Died	Blood	IV	1	1
4	02/2001	98	F	Bacteremic pneumonia	Chronic heart disease	Died	Blood	IV	1	1
5	12/2001	33	F	Meningitis	None	Survived	CSF	I	1	1

^a^Hie, *Haemophilus influenzae* type e; CSF, cerebrospinal fluid; PFGE, pulsed-field gel electrophoresis; M, male; F, female.

By PCR, all isolates exhibited the type e capsular genotype. By slide agglutination method, performed at the national reference laboratory, four isolates were designed as type e, and one was misidentified as nontypable. At the regional level, only one of the five PCR-positive strains had been recognized as type e, two had been identified as nontypable, and two had not been typed. By biotyping, four isolates were classified as biotype IV, and one as biotype I (patient no. 5) (Table 2). MICs of ampicillin ranged from 0.125 µg/mL to 0.25 µg/mL, indicating that all isolates were susceptible to this antibiotic; none produced β-lactamase. As assessed by disk diffusion assay, all isolates were also susceptible to trimethoprim/sulfamethoxazole, ceftazidime, chloramphenicol, azithromycin, aztreonam, ciprofloxacin, imipenem, and tetracycline. PFGE with *Sma*I restriction enzyme digestion generated not well-resolved profiles, as several very close fragments of 194–145 kb were obtained (data not shown). Following *Apa*I digestion, profiles were easier to compare (Figure): three isolates (patients no. 3, 4, and 5) shared an indistinguishable pattern (pattern 1); one isolate (patient no. 1) showed a profile closely related to pattern 1 but with two band differences (pattern 1a). The isolate from patient no. 2 appeared clearly different from the others (pattern 2), according to criteria reported by Tenover et al. (*10*) (Table 2). All Hie isolates were unrelated to invasive Hib strains circulating in Italy (*9*) (Figure). According to epidemiologic data, patients with strains that showed an indistinguishable PFGE pattern did not appear to share any common risk factor. Southern hybridization with the probe for the *cap* e gene after PFGE identified a single fragment of 20.5 kb in each isolate tested, suggesting the presence of one copy of the *cap* locus (Table 2).

**Figure:**
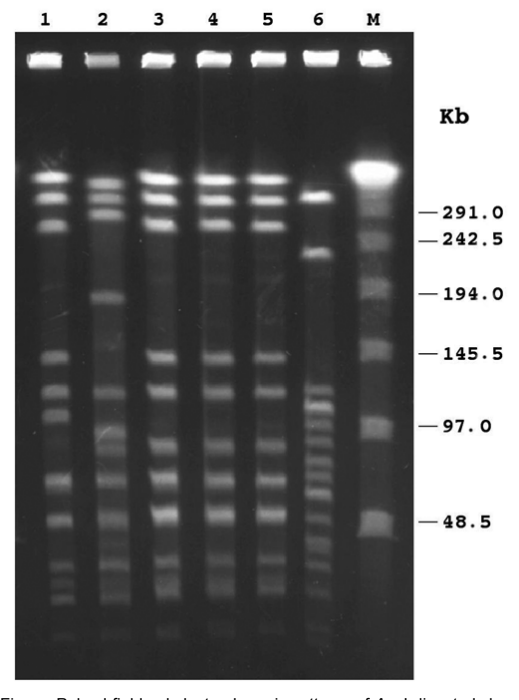
Pulsed-field gel electrophoresis patterns of *Apa*I-digested chromosomal DNAs of *Haemophilus influenzae* isolates. Lanes 1–5, *H. influenzae* type e (Hie) isolates, respectively, from patient nos. 1–5; lane 6, *H. influenzae* type b (Hib) strain belonging to one of the subclones endemic in Italy (9); M, λ ladder pulsed-field gel marker with molecular weights indicated in kilobases (kb) at the right. The isolates in lanes 3, 4, and 5 showed indistinguishable profiles (pattern 1); the isolate in lane 1 was closely related to pattern 1 (pattern 1a), whereas the isolate in lane 2 was clearly different from pattern 1 (pattern 2). All Hie isolates were unrelated to the Hib strain.

## Conclusions

Although studies on nasopharyngeal carriage of *H. influenzae* have shown the presence of serotype e as colonizer (*11*–*13*), few studies have described serious infections attributable to this capsular type (*14**–**17*). Our results suggest that Hie may cause either fatal infections in elderly patients with underlying disease or meningitis in adults whether or not underlying conditions are present. In Italy, these cases of Hie infection are the first reported. Despite limited data on the frequency of non–type b invasive disease before 1997, our results are based on 4 years of laboratory-based active surveillance on a large population sample. Since no type e strains were detected in the first 2 years of this surveillance, this clustering is likely due to the emergence of invasive Hie disease. Alternative explanations might include improved laboratory confirmation methods (however, the proportion of meningitis cases without an identified etiologic agent did not decrease from 1998 to 2001) or improved reporting in the last 2 years of surveillance (however, no changes to the system were made, and quality indicators, such as proportion of isolates sent to the reference laboratory, remained stable throughout the 4 years).

An unequivocal method of assigning capsular type is required to monitor infection attributable to uncommon serotypes, especially since, as in some Hie isolates, expression of capsular polysaccharide is not sufficient to be detected by slide agglutination (*18*). In our study, PCR capsular typing allowed the identification of an additional Hie strain that had been misidentified by slide agglutination.

The prevalence of ampicillin resistance in both β-lactamase–positive and –negative *H. influenzae* isolates has increased worldwide. Neither β-lactamase production nor intrinsic resistance was detected among our Hie isolates, confirming the low incidence of ampicillin-resistant *H. influenzae* strains from invasive disease in Italy (*5*,*9*).

PFGE has been successfully used to type *H. influenzae* isolates. Our results suggest that two distinct Hie strains circulated in Italy in 2000–2001. Four of the five isolates, found in two neighboring regions, appeared to represent a unique clonal group with two subtypes. This clustering of most strains in one PFGE pattern might be explained with the clonal population structure of encapsulated *H.* *influenzae* previously observed in Italy (*5*,*9*), but further studies are needed to clarify this point. Moreover, since the type e isolates analyzed in this study had no close genetic relationship to the invasive Hib strain circulating in Italy, their derivation from type b isolates by capsular switch is unlikely. *H. influenzae* PFGE typing has a stronger discriminatory power than biotyping (*19*). In our study, however, in the isolates assigned to the same clonal group by PFGE, one was classified as biotype I, and the others were biotype IV. These findings suggest that biotyping could be a useful adjunct to PFGE when typing Hie isolates.

In our study, all five Hie isolates contained a single copy of the *cap* locus, suggesting that they did not possess unusual virulent traits related to the capsule. Since the size of the restriction fragment obtained was smaller than expected on the basis of *cap* b locus size, a possible explanation may be that the size of region 2 of *cap* e locus is smaller than that found in Hib strains. Alternatively, the DNA flanking the *cap* e locus may differ from that found in Hib strains; therefore, the segments upstream and downstream from the *cap* e region would be smaller.

Both the laboratory-based active surveillance of invasive *H. influenzae* disease and PCR capsular genotyping could have improved the capability to detect cases attributable to serotypes other than b. Nevertheless, our data suggest the emergence of invasive Hie disease among the adult population in Italy and underline the need to closely monitor infection caused by non–type b strains.
